# Having a latrine facility is not a guarantee for eliminating open defecation owing to socio-demographic and environmental factors: The case of Machakel district in Ethiopia

**DOI:** 10.1371/journal.pone.0257813

**Published:** 2021-09-30

**Authors:** Abathun Temesgen, Mesafint Molla Adane, Amsalu Birara, Tebkew Shibabaw

**Affiliations:** 1 Debre Elias District Health Office, Amhara Region, Ethiopia; 2 Department of Environmental Health, Bahir Dar University, Bahir Dar, Ethiopia; Shahrood University of Technology, ISLAMIC REPUBLIC OF IRAN

## Abstract

**Background:**

Open defecation practice problem is rampant in most rural areas of developing countries, including Ethiopia. To combat this problem, the Ethiopian government implemented different sanitation interventions including Community-Led Total Sanitation and Hygiene (CLTSH). The CLTSH approach is mainly aimed to eradicate open defecation practice through mobilizing the community to construct a latrine facility and utilize it. Although this intervention has significantly improved households’ access to a latrine facility, its impact on bringing behavioral change such as avoiding open defecation is not well studied.

**Objective:**

Our study aimed to assess the prevalence of open defecation among households having their latrine and its determinant factors in rural settings in Northwest Ethiopia.

**Methods:**

A community-based cross-sectional study was conducted in Machakal district from September 1 to 30, 2019. A total of 472 household heads who had a latrine facility and systematically selected from six rural Kebeles of the district, were involved in the study. The data were collected using a structured questionnaire and observational checklist tools through face-to-face interviews and observation methods. Bivariate and multivariable logistic regression models were run to identify the factors that influence open defecation practice. During the multivariable analysis, statistical significance was declared at the p-value of <0.05 with 95% CI.

**Results:**

The prevalence of open defecation practice among household heads who had latrine facility was 27.8% (95% CI, [23.1–32.8]). Female gender (AOR = 2.94, 95% CI [1.13–7.68]), not attending of formal education (AOR = 3.10, CI 95% [1.34–7.13]), having >5 family members (AOR = 1.72, CI 95% [1.05–2.80]), presence of under-five child (AOR = 3.64 CI 95% [2.14–6.21]), preferring leaf as anal cleaning material (AOR = 3.18, CI 95% [1.67–6.08]), having unclean latrine (AOR = 2.15, CI 95% [1.34–3.44]), and having latrine that needs maintenance (AOR = 2.50 CI 95% [1.52–4.11]) variables were associated with open defecation practice.

**Conclusions:**

Among the total respondents, finding more than a quarter of open defecators is concerning for a district that achieved greatly in terms of latrine coverage. This indicates the above-mentioned factors contributed to influence household heads to defecate openly despite having latrines. Therefore, the government and partners need to focus on designing strategies that effectively address determinant factors of open defecation.

## Introduction

Poor sanitation is one of the challenges that hinder a country from ensuring sustainable development by affecting public health, social wellbeing, and the economy [1, 2]. Looking at the public health impact alone, worldwide about half a million diarrheal cases occurring each year are related to poor sanitation [3]. Besides, it is the main cause of many neglected tropical diseases and malnutrition public health threats. The magnitude of sanitation-related public health problems could even be higher in developing countries where poor sanitation practices like open defecation are rampant [1, 3]. According to the World Health Organization (WHO) 2017 report, poor sanitation is responsible for about 30% of annual diarrheal deaths in low-and middle-income countries in which most are under-five children [3].

According to WHO estimates, 673 million people still defecate in open fields around the world [3]. The problem of open defecation practice is more severe in most developing countries like Ethiopia where access to basic sanitation systems is still low [4]. The Ethiopian Demographic and Health Survey (EDHS) 2016 report indicated that 32% of the population practices open defecation of which 7% are urban and 39% are rural communities [5]. As a measure to improve the hygiene and sanitation status of the population, the Ethiopian government has been engaging in various sanitation and hygiene intervention activities [6]. With the support of its development partners, the government has been working relentlessly and implemented different programs/activities to construct sanitation facilities and bring behavioral change to the community. Among the measures taken, the core was the introduction of Community-Led Total Sanitation and Hygiene (CLTSH) in 2006 and its formal adoption by the Federal Ministry of Health (FMoH) in 2011 [7]. The CLTSH approach is mainly aimed to eradicate open defecation practice through mobilizing the community to construct its latrine and utilize it [8]. This effort has brought a positive outcome and since then many districts of the country have graduated to be open defecation-free. However, recent survey reports indicated that households that have access to latrines still practice open defecation due to various factors [9, 10]. Hence, identifying these risk factors and design sustainable strategies is essential to avert the undesirable public health, social, and economic consequences of open defecation.

Previous findings showed that access to a latrine facility is not a guarantee for avoiding open defecation practice [9–16]. Although users have latrines, they still may choose to defecate openly due to socio-demographic, behavioral, cultural, and environmental factors [9, 14, 16]. Factors such as latrine duration, latrine maintenance condition, presence of an under-five child, family income, latrine cleanliness, latrine distance from the living room, water availability, personal beliefs, family size, and exposure to latrine utilization promotional messages were associated with latrine utilization status of users [10, 12, 13, 15, 17].

The Machakal district in the Amhara region is one of the areas in Ethiopia where CLTSH and other sanitation and hygiene interventions have been conducting. According to the 2019 annual report, latrine coverage of the district was 97%. Despite the efforts made by the government and collaborators, attaining and sustaining the “open defecation free” status in the district is still challenging. Some households own latrines but still defecate in open places [18]. Therefore, our study is aimed to determine the magnitude of open defecation practice among households having latrines and identify the factors associated with this behavior. This is important to figure out the main obstacles of latrine utilization and design appropriate intervention strategies accordingly.

## Materials and methods

### Study area

The study was conducted in Machakle district, which is located approximately 328 Km far from the capital of Ethiopia (Addis Ababa) in the northwest direction. It is one of the member districts in East Gojjam Zonal administration in the Amhara National Regional State. According to the 2007 census, the district has a total projected population of 143,516 with 33,376 households. The district has 32 Kebeles (smallest administrative unit in Ethiopia) of which 26 are rural and the remaining 6 are urban. According to the district’s 2019 report, about 97% of the households in the district own latrines [18].

### Study design and period

A community-based cross-sectional study design was carried out from September 1 to 30, 2019.

### Population

The source population of the study was all rural households who had a latrine in the Machakel district. Besides, our study population was all households who had a latrine in six rural Kebeles of the district during the survey. Moreover, household heads selected systematically for the study were our study unit.

### Inclusion and exclusion criteria

All households who resided in the selected Kebeles of Machakel district and had a latrine were included in the study. However, household heads who were severely ill and unable to communicate were not eligible for the survey.

### Sample size determination and sampling procedure

We employed a single population proportion formula to estimate the required number of study participants.


$$n=((Z_{\alpha /2}{)}^{2}\times p(1‐p))/d^{2}$$

We used an open defecation prevalence of 16.9% found from a previously conducted study in Southern Ethiopia [9]. In addition, we assumed a 95% confidence level, a margin error of 5%, a design effect of 2, and a 10% non-response rate. Accordingly, our final sample size was 476.

A multistage sampling procedure was employed to select the study participants. Since we believe the magnitude of open defecation practice is similar across all rural Kebeles in the district is similar, six rural Kebeles were selected randomly for the study. Then we took the list of households that had a latrine in each Kebele from Health Extension Workers and the number of households to be taken from each Kebele was proportionally allocated. Finally, we employed a systematic sampling technique to select the study households since we had a list of households having a latrine from each Kebele.

### Study variables

Open defecation practice was our dependent variable. On the other side, socio-demographic characteristics of households and respondents, environmental conditions (latrine type, latrine condition, living room to latrine distance, latrine cleanliness, availability of handwashing facility), and behavioral factors (anal cleaning material preference, getting of latrine utilization promotional messages, the attitude of respondents) were the independent variables of the study.

### Data collection method and instrument

Data were collected through face-to-face interviews and observation methods. The socio-demographic and behavioral variables data were collected through an interview using a structured questionnaire. In addition, the data collectors observed the environmental conditions of each participant’s latrine using a checklist. Four Environmental Health Professionals who hold bachelor’s degrees and have data collection experience collected the data.

### Data quality control

To reduce inconsistency between data collectors, the data collection tools were prepared ahead of the data collection time and training was provided for them. After training, a pre-test was conducted on 5% of participants selected from Kebeles not belong to the six selected Kebeles for the study. This is important for checking the applicability of data collection procedures and tools. The principal investigator closely supervised data collectors during the data collection period. The collected data were checked for completeness, consistency, accuracy, and clarity each day after data collection.

### Data analysis and presentation

After the data were collected, the principal investigator checked, coded, and entered the data into Epi info and exported it afterward to SPSS (Statistical Package for Social Science) version 21 software for further treatment. To identify predictors of open defecation, we used binary logistic regression models. First, we conducted binary logistic regressions to select the candidate explanatory variables for the multivariable analysis. Independent variables that had a p-value of <0.2 were included in the multivariable analysis and an odds ratio at 95% CI was used to measure the strength of association between outcome and predictor variables. Those predictors with a p-value of <0.05 were labeled as significantly associated with open defecation practice. The goodness of fit of the models was assessed using the Hosmer-Lemeshow test. Finally, we used frequency tables to present the results.

### Ethics statement

Before collecting the data, an ethical clearance letter was obtained from Bahir Dar University, College of Medicine and Health Sciences Ethical Review Board. In addition, we received legal permission with a letter of support from the Machakel District Health Office. Before data collection, verbal and written consent was obtained from each study participant after the data collectors explained the purpose of the study.

### Operational definition of terms

- **Open defection practice**—is behavior and we operationalized it when the respondent (household head) admits that he/she defecates on open fields irrespective of its frequency.
- **Functional latrine–**is a latrine that provides service at the time of data collection.
- **Latrine condition**–is the state of the latrine that describes whether it needs urgent maintenance or not. We labeled the latrine, as “needs maintenance” if one or more of the latrine components is broken or collapsed. The components considered are; the squatting hole, the slab, the wall, roof, window, and door.
- **Clean latrine**–is when there is no fecal matter inside the facility *i*.*e*., visible on the door, floor, or wall.
- **Attitudes towards OD practice:** It is individual belief on open defecation practice and latrine utilization. We used 5 questions to measure it. Then we used a 5-point Likert-scale measurement to categorize each respondent’s attitude as “favorable” or “unfavorable”. Respondents were labeled as having a “favorable” if their response was ≥ the median score of attitude questions otherwise they were labeled as having an “unfavorable” attitude towards OD practice.

## Results

From a randomly selected six Kebeles of Machakal district, 474 household heads participated in the present study with a response rate of 99.6%.

### Socio-demographic characteristics

Of the total respondents, 399 (84.2%) were males and the remaining 75 (15.8%) were females. Regarding marital status, 371 (78.3%) were married followed by divorced those accounts for 49 (10.3%). The mean age of respondents was 46.1 years with a standard deviation of 13.4 years. Four hundred (84.4%) of the respondents did not attend formal education and 416 (87.8%) were farmers. Among the households, 267 (56.3%) had above 5 family members and 355 (74.9%) had at least a child who was attending formal education as shown in Table 1.

**Table 1:** Socio-demographic characteristics of respondents of study households.

Variables		Frequency	Percentage
**Sex**	Male	399	84.2
	Female	75	15.8
**Age**	≤35	129	27.2
	36–55	246	51.9
	<55	99	20.9
**Marital status**	Single	24	5.1
	Married	371	78.3
	Divorced	49	10.3
	Widowed	30	6.3
**Educational status**	Not attended formal education	400	84.4
	Attended formal education	74	15.6
**Occupation**	Farmer	416	87.8
	Merchant	22	4.6
	Daily laborer	36	7.6
**Family size**	≤5	207	43.7
	>5	267	56.3
**Presence of under-five child**	No	175	36.9
	Yes	299	63.1
**Presence of child attending formal education**	No	199	25.1
	Yes	355	74.9

### Household latrine characteristics

Regarding latrine type, 442 (93.2%) of households had pit latrine without slabs. During the survey, 436 (92.0%) of the latrines were functional and 401 (84.6%) had superstructure. Of the available latrines we observed, 238 (50.2%) were clean, 332 (70.0%) had no squatting hole cover, and 268 (56.5%) need maintenance. Besides, 309 (65.2%) latrines were constructed six and above meters far from the living room, 278 (58.6%) had no water storage material inside, and 425 (89.7%) had no any form of handwashing facility (Table 2).

**Table 2:** Latrine characteristics and availability of handwashing facility of study households.

Variables		Frequency	Percentage
**Type of Latrine**	Pit latrine without a slab	442	93.2
	Pit latrine with a slab	27	5.7
	VIP	5	1.1
**The functionality of the latrine**	Not functional	38	8
	Functional	436	92
**Latrine sharing with other households**	No	419	88.4
	Yes	55	11.6
**Distance between the living room and latrine (meter)**	<6	165	34.8
	≥6	309	65.2
**Presence of superstructure**	No	73	15.4
	Yes	401	84.6
**Cleanliness of the latrine**	Not clean	238	50.2
	Clean	236	49.8
**Presence of cover for squatting hole**	No	332	70
	Yes	142	30
**Latrine condition**	Does not need maintenance	206	43.5
	Needs maintenance	268	56.5
**The main source for flushing/hand washing**	Piped water	43	9.1
	Well	39	8.2
	Hand pump	153	32.3
	Stream and river	239	50.4
**Distance between water source and latrine (meter)**	<10	94	19.8
	≥10	380	80.2
**Presence of water storage facility inside the latrine**	No	278	58.6
	Yes	196	41.4
**Presence of hand washing facility**	No	425	89.7
	Yes	49	10.3

### Behavioral factors

From the household heads who participated in the study, 27.8% (95% CI, [23.1–32.8]) responded that they practiced open defecation despite having a latrine facility. Fear of collapse of latrine slab (similar structure placed on the pit) was the main reason (36.1%) for practicing open defecation followed by having a big squat hole of the latrine. Two hundred ninety-one (61.4%) of the participants had a favorable attitude towards open defecation. Regarding anal cleaning material preference, 246 (51.9%) of the respondents prefer leaf followed by water which accounts for 28.1%. In addition, 276 (58.2%) of the participants responded that they did not see/hear a single message that promotes latrine utilization in the past year (Table 3).

**Table 3:** Behavioral conditions of respondents.

Variables		Frequency	Percentage
**Practice open defecation**	No	342	72.2
	Yes	132	27.8
**Reason for open defecation**	Big squat hole of latrine	114	28.8
	Offensive odor	113	28.5
	Fear of collapse of latrine slab	143	36.1
	Latrine lacks superstructure	26	6.6
**Attitude towards OD**	Favorable	291	61.4
	Unfavorable	183	38.6
**Preferred anal cleaning material**	Paper/tissue paper	95	20
	Leaf	246	51.9
	Water	133	28.1
**Seen/heard any latrine utilization promotional message during the last year**	No	276	58.2
	Yes	198	41.8

### Factors affecting open defecation practice

The multivariable logistic regression results presented in Table 4 indicate that sex, educational status, family size, and presence of under-five child family members were the socio-demographic predictors of open defecation in the study area (p<0.05). Besides, anal cleaning material preference, latrine cleanliness, and latrine condition were the environmental and behavioral factors associated with open defecation practice (p<0.05).

**Table 4:** Logistic regression analysis results showing predictors of open defecation.

Variables		Practice open defecation		AOR (95% CI)
		No	Yes	
**Sex**	Male	295	104	1
	Female	47	28	2.94 (1.13–7.68) *
**Age (in years)**	≤35	101	28	0.50 (0.24–1.04)
	36–55	173	73	0.74 (0.40–1.34)
	<55	68	31	1
**Marital status**	Single	18	6	1.58 (0.36–6.88)
	Married	275	96	1.17 (0.37–3.49)
	Divorced	30	19	1.15 (0.38–3.49)
	Widowed	19	11	1
**Educational status**	Not attended formal education	277	123	3.10 (1.34–7.13)**
	Attended formal education	65	9	1
**Family size**	≤5	162	45	1
	>5	180	87	1.72 (1.05–2.80) *
**Presence of under-five child**	No	147	28	1
	Yes	195	104	3.64 (2.14–6.21)**
**Presence of school child**	No	79	40	1.53 (0.89–2.64)
	Yes	263	92	1
**Anal cleaning material preference**	Paper/tissue paper	68	27	3.13 (1.48–6.61)**
	Leaf	164	82	3.18 (1.67–6.08)**
	Water	110	23	1
**Got latrine utilization promotion message**	No	187	89	1.62 (0.99–2.67)
	Yes	155	43	1
**Functionality of latrine**	No	24	14	1.45 (0.62–3.38)
	Yes	318	118	1
**Presence of superstructure**	No	46	27	1.09 (0.57–2.08)
	Yes	296	105	1
**Presence of water storage facility**	No	192	86	1.41 (0.86–2.32)
	Yes	150	46	1
**Cleanliness of the latrine**	Not clean	154	84	2.15 (1.34–3.44)**
	Clean	188	48	1
**Latrine condition**	Needs maintenance	170	98	2.50 (1.52–4.11)**
	Does not need maintenance	172	34	1
**Distance between the living room and latrine (m)**	<6	129	36	0.63 (0.38–1.04)
	≥6	213	96	1

* = significantly associated at p<0.05

** = significantly associated at p<0.01.

In this study, women were more likely to defecate in open fields (AOR = 2.94, 95% CI [1.13–7.68]) than men. Household heads who did not attend formal education were 3.10 times more likely to practice open defecation (AOR = 3.10, CI 95% [1.34–7.13]) than their counterparts. The odds of respondents with above five family members to practice open defecation were more than (AOR = 1.72, CI 95% [1.05–2.80]) respondents who had five and below family size. Additionally, households who had at least one under-five child member were 3.64 more likely to practice open defecation (AOR = 3.64 CI 95% [2.14–6.21]) than households that did not have (Table 4).

When it comes to behavioral factors, the odds of practicing open defecation were 3.18 times higher in respondents who prefer leaf to clean their anus (AOR = 3.18, CI 95% [1.67–6.08]) than participants who prefer to use paper/tissue paper. Regarding the environmental factors, households that had unclean toilets were 2.15 times more likely to defecate in open spaces (AOR = 2.15, CI 95% [1.34–3.44]) than households who had clean latrines. Additionally, families who had a latrine in a condition that needs urgent maintenance were more likely to practice in open fields (AOR = 2.50, CI 95% [1.52–4.11]) than those who had a latrine in a good condition as presented in Table 4.

## Discussion

Our study revealed that the magnitude of open defecation practice among households having a latrine was 27.8% (95% CI, [23.1, 32.8]). Our study was similar to earlier studies conducted in Nigeria (24%) and India (23.2%) in which communities in the rural places practiced open defecation [19, 20]. However, this finding was inconsistent with previous studies done in Ethiopia [9, 10]. The study conducted in Wondo Genet district in South Ethiopia showed only 16.9% of the households did not use latrines [9]. The possible reason for the discrepancy could be differences in the study period between the studies might affect the sustainability of CLTSH implementation [21]. The other reason could be differences in the educational status of respondents in which only 15.6% attended formal education in our study compared to 47.4% in Ashenafi *et al*.*’s* study. A systematic review and meta-analysis study indicated that the educational status of respondents in Ethiopia was important in influencing latrine utilization behavior [22]. Contrarily, we found lower open defecation prevalence compared to a study by Oljira & Berkessa (64%) conducted in Ilu Ababor Zone in Southwest Ethiopia [10]. This might be due that the CLTSH intervention was implemented in our study Kebeles that could change the behavior of individuals towards using a latrine [8]. Another possible influencer would be the study time difference between the studies. The finding in this study was also different from a study done in rural South India (54.8%) among households that had latrine facilities [16]. This discrepancy might arise from differences in the number of study participants involved in one household. We determined the behavior of open defecation if it was practiced by the respondent only. Contrarily, the study from South India accounted for the OD practice of all household members. This implies there could be another family member who could defecate openly even if the respondent did not practice and this definitely would increase the magnitude. Another possible explanation for the variation could be differences in socio-demographic characteristics and cultural background of respondents, environmental conditions, and intervention modalities between the studies.

Previous studies conducted in Ethiopia [9, 10, 12] and other developing countries [11, 13, 16, 19] showed the role of socio-demographic, cultural, behavioral, and environmental factors in influencing households to practice open defecation despite having latrine. Individual characteristics (gender, educational status) of respondents are among the main factors that influence open defecation practice [11, 22]. The odds of open defecation practice by females were higher than males. This finding is supported by a qualitative study done in rural Nepal in which females were forced to go for open defecation as female family members are not allowed to utilize the latrine at home [14]. However, there is no apparent culture that restricts females to not utilize a latrine in the study district. Higher open defecation practice by women heads in our study might be related to taking more responsibility to take care of their under-five children. In rural parts of Ethiopia, there is a habit of defecating in open fields by some mothers or caregivers while they assist their baby to defecate. This is also supported by the multivariable analysis in which the open defecation practice of respondents was associated with the presence of an under-five child family member in the household. The study also confirmed that household heads who did not attend formal education were at high risk of practicing open defecation compared to their counterparts. Studies conducted in Ethiopia [22] and Timor-Leste [13] supported our findings. Education influences human behavior towards practicing healthy activities [23]. Hence, latrine utilization practice improves with an increase in the educational level of individuals as they would be more aware of the disease burden of poor sanitation practices.

The likelihood of practicing open defecation by individuals who prefer leaf as anal cleaning material was higher than individuals who prefer to use water and paper/tissue paper. This is linked to the reason that finding leaf materials in bushes, riverbanks, and other outdoor areas would be more convenient than in latrines. This promotes individuals who prefer leaf as anal cleaning material to defecate in open fields/bushes. Users prefer to clean their anus with materials that are easily accessible during defecation [24].

Family dynamics also influenced the open defecation practice of household heads in the study area. We confirmed that having more family members in the household was the risk factor for open defecation. Our finding agreed with earlier studies conducted in Ethiopia [25] and Ghana [26]. Households that have more members could be forced to go out for defecation due to overcrowding particularly during morning hours [16]. Similar to studies by others [9, 17], the presence of an under-five child member in the house was another encouraging factor for open field defecation. The possible justification could be that under-five children are allowed to defecate openly since latrines are not convenient for them. During defecation, the children are mostly accompanied by older family members (mainly mothers) and this could encourage the caretaker to practice open defecation as well.

Earlier studies reported in Ethiopia and elsewhere pinpointed the role of environmental factors in influencing the latrine utilization practice of respondents [9, 12, 15, 16]. In this study, household heads with unclean latrines were more likely to practice open defecation compared to those that had clean latrines or clean more frequently. Previous studies reported in Ethiopia [15, 25] agreed with our finding. Latrines contaminated with excreta and other filthy matters discourage users to utilize the toilet [14]. Similarly, a latrine that is in bad condition or needs maintenance cannot attract users due to privacy and security reasons. People could develop a fear of collapsing, lack privacy, and other security issues if the physical structures like squatting holes, slabs, doors, walls, and roofs are damaged and need maintenance [16]. In our study, needing maintenance of the latrine was identified as the risk factor for practicing open defecation among the respondents and this agreed with previous studies conducted in Ethiopia [9, 10].

The prevalence of diarrheal diseases in under-five children in Ethiopia ranged from 9.9 to 17.2% in villages that declare open defecation-free (ODF) status and 23.2 to 36.3% in villages that practice open defecation [27–29]. This indicates the role of tackling open defecation practice in promoting public health and achieving sustainable development goals 2030 is enormous. This study showed socio-demographic, latrine-related, and behavioral factors restricted over a quarter of household heads from using their latrine facility. This implies understanding the nature of these factors and considering practical solutions is pivotal for formulating environmental health policy and implementing sanitation intervention programs that target eliminating open defecation in the study district as well as Ethiopia. For instance, the provision of health information that promotes latrine usage by the district health office authorities is important to resolve education and gender-related hurdles. Besides, incorporating sanitation in school curricula for primary and secondary school students and running Environmental Health clubs in schools could be instrumental to bring behavioral change among school children. This strategy could be more effective in bringing massive behavioral change in the community towards using latrine as school children could serve as change agents by transmitting the information they gained at school to their family as well as neighbors. In addition, balancing latrine facility to family size ratio by constructing additional latrine facilities could be crucial to reduce open defecators in households with more family members. For this, district health offices can deploy health extension workers to identify households that have insufficient latrine facilities (households with low latrine to family size ratio) through house-to-house surveys and assist these households to construct additional latrine facilities. In addition, considering the inclusiveness of the facility while constructing and monitoring this by public health workers (health extension workers) is essential to accommodate all family members including under-five children.

Since its introduction into Ethiopia, the CLTSH intervention has brought a significant change in increasing latrine coverage and reducing open defecation in the country. However, open defecation slippage of ODF-certified villages in different areas of the country is reported in recent years. This slippage is mainly due to lack of technical support, poor-quality construction material, lack of follow-up, financial constraints, improper implementation of the CLTSH program, and demotivation due to the poor-quality latrine [30]. Our study also indicates poor conditions of latrines (unclean latrine and need maintenance) were the factors that promote household heads to practice open defecation. This implies providing technical support during latrine construction and sustained follow-up by local authorities is necessary to consult the community to clean their latrine regularly and maintain the latrine when it needs. Besides, public health workers and other actors of sanitation should; educate the community about the benefits of latrine use, assess the levels of social belief, and assist in latrine technology selection and handling to enhance latrine utilization. Finally, the existing National Sanitation and Hygiene Strategy of Ethiopia should include post-ODF programs that focus on sustainable latrine utilization.

Although we utilized maximum efforts to maintain the quality of the study, some factors may affect the quality of our findings. The nature of the study design (cross-sectional) could affect the temporal relationship between some predictors and the response variable. The social desirability bias of respondents could be another factor affecting self-reporting latrine use despite non-usage.

## Conclusions

Despite having a latrine facility, over a quarter of study participants practiced open defecation. This behavior was influenced by socio-demographic, behavioral, and environmental factors. The study identified elements such as being female, not attending formal education, having more than five family members, presence of an under-five child, preferring leaf as anal cleaning material, having unclean latrine, and having latrine that needs maintenance as risk factors of open defecation practice. This indicates having a latrine is not a guarantee for eradicating open defecation practice. Therefore, to curb the problem of open defecation, the government at all levels, NGOs, and other actors engaged in sanitation intervention activities need to focus on designing strategies valid to address contributing factors of open defecation. Moreover, the findings of this study have important implications for environmental health programs and can be used to formulate policies towards addressing open defecation in Ethiopia, and other developing countries.

## Supporting information

S1 File Data collection tool. (PDF)

S2 File Data. (XLSX)
