# Supplementary material for: Having a latrine facility is not a guarantee for eliminating open defecation owing to socio-demographic and environmental factors: The case of Machakel district in Ethiopia
Source: PLoS One. 2021 Sep 30;16(9):e0257813. doi: 10.1371/journal.pone.0257813 (PMC8483416; doi:10.1371/journal.pone.0257813)
Supplement: S1 File — (PDF) [file pone.0257813.s001.pdf]

Data collection tool – English version

This tool is designed to assess prevalence of open defecation practice and its determinant factors in Machakel district, East Gojjam Zone, Amhara region, Ethiopia, 2019.

- Date of interview -------------------------
- Questionnaire number--------------------
- Sub-location (village) name -------------
- Respondent code ------------------------

1. Questionnaire

Part I: Socio-demographic and economic characteristics				
	**Questions**	**Alternatives**	**Code**	**Skip**
101	Gender	Male<br>Female	1<br>2	
102	Age (in year)	---------		
103	Marital status	Single<br>Married<br>Divorced<br>Widowed	1<br>2<br>3<br>4	
104	Education status	Illiterate<br>Primary education<br>Secondary education<br>Above secondary education	1<br>2<br>3<br>4	
105	Occupation	Farmer<br>Merchant<br>Daily laborer<br>Government employee<br>Self-employee<br>Other---------	1<br>2<br>3<br>4<br>5<br>99	
106	Family size	------------		
107	Do you have under-five child in the house?	Yes<br>No	1<br>2	
108	Is there a child attending formal education in the house?	Yes<br>No	1<br>2	
109	If your answer for Q#108 is Yes, what is the level of their education	Primary education<br>Secondary education<br>Higher education	1<br>2<br>3	

	**Part II: Behavioral factors**			
201	Do you practice open defecation?	Yes<br>No	1<br>2	
202	If yes Q #201, where do you defecate?	In agricultural fields<br>Near water sources<br>In bushes/forests<br>Other----------	1<br>2<br>3<br>99	
203	If yes for Q #201, how frequent do you practice it?	Always<br>Mostly<br>Sometimes<br>Rarely	1<br>2<br>3<br>4	
204	Reason for practicing open defecation	-------------------------		
205	Which material do you prefer to clean your anus after defecation?	Paper/tissue paper<br>Leaf<br>Water<br>Sediment or stone<br>Other-----------	1<br>2<br>3<br>4<br>99	
206	How do you usually dispose of baby's feces? (Circle only one which is very often)	Put into latrine using Popo<br>Put into drain/ditch<br>Put together with garbage<br>Burry<br>Left on open fields<br>Other ------------	1<br>2<br>3<br>4<br>5<br>99	
207	Have you seen/heard any latrine utilization promotional message during the last one year?	Yes<br>No	1<br>2	
208	If yes, through which source or media have you seen/heard? (Circle all that apply).	Radio /Television<br>Newspaper<br>Health Extension Worker<br>Developmental army<br>Family member<br>Others ---------	1<br>2<br>3<br>4<br>5<br>99	
	**Part III. Attitude towards open defecation**			
301	Open defecation practice is a good habit and should be continued	Strongly disagree<br>Disagree<br>Neutral<br>Agree<br>Strongly agree	1<br>2<br>3<br>4<br>5	
302	Defecating in open field is not embarrassing	Strongly disagree<br>Disagree<br>Neutral<br>Agree	1<br>2<br>3<br>4	

		Strongly agree		
303	Defecating near surface water sources has no problem	Strongly disagree<br>Disagree<br>Neutral<br>Agree<br>Strongly agree	1<br>2<br>3<br>4<br>5	
304	Defecating in open fields/bushes is more comfortable than using latrine	Strongly disagree<br>Disagree<br>Neutral<br>Agree<br>Strongly agree	1<br>2<br>3<br>4<br>5	
305	Open defecation practice put the community at risk of contracting a disease	Strongly disagree<br>Disagree<br>Neutral<br>Agree<br>Strongly agree	1<br>2<br>3<br>4<br>5	
	Part IV: Latrine characteristics			
401	Is your latrine Functional?	Yes<br>No	1<br>2	
402	When was your latrine constructed?	------------		
403	Do you share the latrine with other households?	Yes<br>No	1<br>2	
404	If yes, how many households share it with you?	-------------		
405	Did you upgrade or reconstruct the latrine?	Yes<br>No	1<br>2	
406	Is there open space suitable for defecation close to your house?	Yes<br>No	1<br>2	
407	What is your main source of water for toilet flushing/hand washing?	Pipe water<br>Well<br>Hand pump<br>Stream/river<br>Pond/lake	1<br>2<br>3<br>4<br>5	

2. Observational checklist

501	Type of toilet facility the household has	Pit latrine without slab<br>Pit latrine with slab<br>VIP latrine<br>Others ……	1<br>2<br>3<br>99	
502	Presence of fresh foot path leading to the latrine and Splash of urine or water on the latrine slab/floor	Yes<br>No	1<br>2	
503	Cleanliness of the latrine	Clean<br>Not clean	1<br>2	
504	Does the latrine need urgent maintenance?	Yes<br>No	1<br>2	
505	Latrine super structure (circle all that apply)	Wall<br>Roof<br>Door<br>Window<br>Pit Slab	1<br>2<br>3<br>4<br>6	
506	Does the latrine have good illumination?	Yes<br>No		
507	Distance of latrine from the living room (meter)	-------		
508	Presence of latrine cover on the squatting hole	Yes<br>No	1<br>2	
509	Distance between water source and latrine?	<10m<br>≥10m	1<br>2	
510	Is water available for toilet use inside/near the latrine?	Yes<br>No	1<br>2	
511	Presence of handwashing facility near the latrine?	Yes<br>No		
512	Presence of water in the hand washing facility?	Yes<br>No		

If there is any question or comment welcome

Thank you for your participation!!!

Data collection tool- Amharic version

በምስራቀ ጎጃም ዞን፤ ማቻክል ወረዳ ማህበረሰብ ዉስጥ ክፍት የመፀዳዳት ልምምድንና ምክኒያቶችን ለመገምገም በተመለከተ ለማጥናት የተዘጋጀ ቃለ-መጠይቅ፡

- የቃለ መጠይቅ የተደረገበት ቀን ---------
- የመጠይቅ ቁጥር ----------------------
- የአካባቢው (መንደር) ስም ----------
- የተሳታፊ ኮድ ---------------------

1. የቃለ-መጠይቅ ቅፅ

ክፍል 1፡ አጠቃላይ ማህበራዊና ስነ-ህዝባዊ መረጃዎችን በተመለከተ				
ተ.ቁ	ጥያቄዎች	አማራጭ	ኮድ	እለፍ
101	ፆታ	ወንድ<br>ሴት	1<br>2	
102	ዕድሜ	-------------		
103	የጋብቻ ሁኔታ	ያላገባች/ባ<br>ያገባች/ባ አብረው የሚኖሩ<br>የተፋታች/ታ<br>የሞተባት/በት	1<br>2<br>3<br>4	
104	የትምህርት ደረጃ	ያልተማረ<br>የመጀመሪያ ደረጃ የተማረ<br>ሁለተኛ ደረጃ የተማረ<br>ከሁለተኛ ደረጃ በላይ	1<br>2<br>3<br>4	
105	ሥራ	ግብርና/ገበሬ<br>ነጋዴ<br>የቀን ሰራተኛ<br>የመንግስት ሰራተኛ<br>የግል ሰራተኛ<br>ሌላ ካለ ይገለጽ------	1<br>2<br>3<br>4<br>5<br>99	
106	ጠቅላላ የቤተሰቡ ብዛት	----------<br>--------		
107	በቤተሰቡ ውስጥ ከአምስት አመት በታች ሆነ ልጅ አለ?	አዎ<br>የለም	1<br>2	
108	ዕድሜያቸው ከየትኛውም የት/ት ዘመን ጀምሮ መደበኛ ትምህርት የሚከታተሉ ልጆች አሉ?	አዎ<br>የለም	1<br>2	
109	አዎ ከሆን የትምህርት ደረጃቸው ምን ያህል ነው?	የመጀመሪያ ደረጃ<br>ሁለተና ደረጃ	1<br>2	

		ዲሎማ እና ከእዚያ በላይ	3	
ክፍል 2፡ ስነ-ባህሪን በተመለከተ ጥያቄዎች				
201	ክፍት ቦታ ይፀዳዳሉ?	አዎ<br>የለም	1<br>2	
202	ለጥያቄ 201 መልስዎ"አዎ" ከሆኑ የት ነዉ የሚፀዳዱት?	የእርሻ ቦታዎች አካባቢ<br>የውሃ ምንጭ አጠገብ<br>በቅርብ /ቁጥቋጦ አጠገብ<br>ሌላ ካለ ይጥቀሱ------	1<br>2<br>3<br>4	
203	ለጥያቄ 201 መልስዎ "አዎ" ከሆኑ መቸ መቸ ነዉ የሚፀዳዱት?	ሁልጊዜ<br>አብዛኛዉን ጊዜ<br>አልፎ አልፎ<br>አንዳንድ ጊዜ	1<br>2<br>3<br>4	
204	ክፍት ቦታ የሚፀዳዱበት ምክኒያት ምንድን ነዉ?	------------------		
205	ከተፀዳዱ በኋላ ለማፅዳት ምንን መጠቀም ይመርጣሉ?	ወረቀት/ሶፍት<br>ቅጠል<br>ዉሃ<br>አፈር/ድንጋይ<br>ሌላ ካለ-----	1<br>2<br>3<br>4<br>5	
206	አብዛኛዉን ጊዜ የህፃናትን ሰገራ እንዴት ነዉ የምታስወግዱት?	በፖፖ ተቀብለን መፀዳጃ ቤት<br>እንጥለዋለን<br>በአጠገብ በሚገኝ ገደላማ ቦታ<br>እንጥለዋለን<br>ከቆሻሻ ጋር እንጥለዋለን<br>ሜዳ ላይ እንተወዋለን<br>ሌላ ካለ----------	1<br>2<br>3<br><br>4<br>99	
207	ባለፈዉ አንድ አመት ዉስጥ ስለመፀዳጃ ቤት አጠቃቀም ሰምተዉ/አይተዉ ያዉቃሉ?	አዎ<br>የለዉም	1<br>2	
208	ለጥያቄ 207 መልስዎ አዎ ከሆነ ከምን ምንጭ/ሚዲያ ነዉ የሰሙት?	ራዲዮ/ቴሌቪዥን<br>ጋዜጣ<br>ከጤና ሰራተኞ<br>ልማት ቡድን መሪዎ<br>ከቤተሰብ አባል<br>ሌላ ካለ-------	1<br>2<br>3<br>4<br>5<br>99	
ክፍል 3፡ አመለካከትን በተመለከተ				
301	ሜዳ ላይ መፀዳዳት ጥሩ ልምድ ስለሆነ፤ሊቀጥል ይገባል፡፡	በጣም አልስማማም<br>አልስማማም<br>ከሁለቱም አይድለም<br>እስማማለሁ<br>በጣም እስማማለሁ	1<br>2<br>3<br>4<br>5	
302	ሜዳ ላይ መፀዳዳት አያሳፍርም/አያሸማቅቅም፡፡	በጣም አልስማማም<br>አልስማማም<br>ከሁለቱም አይድለም	1<br>2<br>3	

		እስማማለሁ<br>በጣም እስማማለሁ	4<br>5	
303	ሜዳ፤ቁጥቋጦ እና ዉሃ አካባቢ መፀዳዳት ምንም ጉዳት የለዉም	በጣም አልስማማም<br>አልስማማም<br>ከሁለቱም አይድለም<br>እስማማለሁ<br>በጣም እስማማለሁ	1<br>2<br>3<br>4<br>5	
304	መፀዳጃ ቤት ዉስጥ ከመጠቀም ይልቅ ሜዳ ላይ መፀዳዳት የበለጠ ምቾት ይሰጣል፡፡	በጣም አልስማማም<br>አልስማማም<br>ከሁለቱም አይድለም<br>እስማማለሁ<br>በጣም እስማማለሁ	1<br>2<br>3<br>4<br>5	
305	ሜዳ ላይ መፀዳዳት ህብረተሰቡን ለበሽታ ይዳርግል፡፡	በጣም አልስማማም<br>አልስማማም<br>ከሁለቱም አይድለም<br>እስማማለሁ<br>በጣም እስማማለሁ	1<br>2<br>3<br>4<br>5	
**ክፍል 4፡ ከመፀዳጃ ቤት ጋር የተያያዙ መጠይቆች**				
401	መፀዳጃ ቤቱ አሁን አገልግሎት ይሰጣል?	አዎ<br>የለም	1<br>2	
402	መፀዳጃ ቤቱ ከተገነባ ምን ያህል አመት ሆነዉ?	-------------		
403	ከሌላ ቤተሰብ ጋር መፀዳጃ ቤቱን አብረዉ ይጠቀማሉ?	አዎ<br>የለም	1<br>2	
404	ለጥያቂ 403 መልስዎ፤ አዎ፤ ከሆነ ከምን ያህል አባዎራወች ጋር?	------------		
405	መፀዳጃ ቤቱ ተጠግኗል/እንደገና ተሰርቷል?	አዎ<br>የለም	1<br>2	
406	ክፈት ቦታ፤ዉሃማ፤ቁጥቋጦ፤ በአካባቢዎ አለ?	አዎ<br>የለም	1<br>2	
407	ለመፀዳጃ ቤቱን ለማጠብ/እጅን ለመታጠብ ዉሃ ከምን ነዉ የምናገኘዉ?	ከቧምቧ<br>ከጉድጓድ<br>ከእጅ ፓምፕ<br>ከወነዝ/ከምንጭ<br>ከኩሬ/ሃይቅ	1<br>2<br>3<br>4<br>5	

2. የምልከታ መጠይቅ

501	ምን አይነት መፀዳጃ ቤት ነዉ ያላቸዉ	ባህላዊ የጉድጓድ ክዳን የሌለዉ<br>ባህላዊ የጉድጓድ ክዳን ያለዉ<br>ቪ.አይ.ፒ መፀዳጃ ቤት<br>ሌላ ካለ---------	1<br>2<br>3<br>99	
502	መፀዳጃ ቤት ጋር የሚያገናኝ አዲስ መንገድ፤ሽንት እና ዉሀ መፀዳጃ ቤቱ ወለል ላይ ይታያል?	አዎ<br>የለም	1<br>2	
503	የመፀዳጃ ቤቱ የንፅና ሁኔታ	ንጹህ ነዉ<br>ንጹህ አይደለም	1<br>2	
504	በአሁኑ ጊዜ መፀዳጃ ቤቱ ጥገና ይፈልጋል?	አዎ<br>የለም	1<br>2	
505	መጸዳጃ ቤቱ ያሉት የላይኛዉ ክፍሎች	ግድግዳ<br>ጣሪያ<br>የሚዘጋ በር<br>የሚሰራ ፒት/ጉድጓድ	1<br>2<br>3<br>4	
506	መፀዳጃ ቤቱ በቂ የሆነ ብርሃን አለዉ?	አዎ<br>የለም	1<br>2	
507	መጸዳጃ ቤቱ ከመኖሪያ ቤት ያለዉ ረቀት (ሜትር)	------------		
508	የመፀዳጃ ቤቱ ጉድጓድ ክዳን አለዉ?	አዎ<br>የለም	1<br>2	
509	መፀዳጃ ቤቱ ከዉሃ መገኛ ቦታ ያለዉ ርቀት?	<10ሜትር<br>≥10ሜትር	1<br>2	
510	መፀዳጃ ቤቱ ወለል ላይ የሚታይ ዉሃ አለ?	አዎ<br>የለም	1<br>2	
511	መታጠቢያ እጅ መታጠቢያ ከመፀዳጃ ቤቱ ጋር ይገኛል?	አዎ<br>የለም	1<br>2	
512	ከእጅ መታጠቢያዉ ጋር ዉሃ አለ?	አዎ<br>የለም	1<br>2	

ጥያቄና አስተያየት ካለዎት መጠየቅ ይቻላል

ስለነበረን ቆይታ እጅግ አመሰግናለው!!!
